# Early Postoperative Expansion of Parenchymal High-intensity Areas on T_2_-weighted Imaging Predicts Delayed Cerebral Edema Caused by Carmustine Wafer Implantation in Patients with High-grade Glioma

**DOI:** 10.2463/mrms.mp.2015-0054

**Published:** 2015-12-28

**Authors:** Yosuke MASUDA, Eiichi ISHIKAWA, Tetsuya YAMAMOTO, Masahide MATSUDA, Hiroyoshi AKUTSU, Hidehiro KOHZUKI, Kei NAKAI, Emiko OKAMOTO, Shingo TAKANO, Tomohiko MASUMOTO, Akira MATSUMURA

**Affiliations:** 1 Department of Neurosurgery, Faculty of Medicine, University of Tsukuba 2-1-1 Amakubo, Tsukuba, Ibaraki 305-8575, Japan; 2 Department of Radiology, University of Tsukuba

**Keywords:** intraoperative magnetic resonance imaging, chemotherapy, high-grade glioma, glioblastoma, BCNU wafer

## Abstract

**Background:**

Carmustine (BCNU) wafer (Gliadel^®^ Wafer) implantation after tumor resection is an approved treatment for high-grade glioma (HGG). These wafers change various characteristics on early postoperative magnetic resonance imaging (ep-MRI) including slight expansion of high-intensity areas on T_2_-weighted imaging (ep-T_2_-HIAs) into adjacent parenchyma without restricted diffusivity. We assessed the frequency of the ep-T_2_-HIAs after BCNU wafer implantation in HGG patients. Moreover, we focused on ep-T_2_-HIA expansion and its relation to delayed cerebral edema.

**Methods:**

Twenty-five consecutive HGG patients who underwent BCNU wafer implantation were assessed. First, patients were divided into ep-T_2_-HIA and non-ep-T_2_-HIA groups, and the incidence of delayed adverse effects was compared between the two groups. Subsequently, the patients were divided into delayed edema and non-delayed edema groups, and pre-, intra-, and postoperative data were compared between the two groups.

**Results:**

The ep-T_2_-HIA expansion and the delayed edema were evident in 9 cases (36%) and 12 cases (48%), respectively. In comparison of the ep-T_2_-HIA and non-ep-T_2_-HIA groups, delayed edema was the only delayed adverse effect associated with ep-T_2_-HIA expansion (*P* = 0.004). Univariate analysis showed a significantly higher ratio of delayed edema in the subgroups with maximal diameter of removed cavity ≤40 mm (*P* = 0.047) and the ep-T_2_-HIA expansion in comparison of the delayed edema and non-delayed edema groups. Multivariate analysis showed that the ep-T_2_-HIA expansion was the only independent factor associated with delayed edema (*P* = 0.021).

**Conclusion:**

In BCNU wafer implantation cases, ep-T_2_-HIA expansion was a predictive factor for delayed cerebral edema.

## Introduction

Carmustine (1,3-bis[2-chloroetyl]-1-nitrosurea (BCNU)) wafer implantation after tumor resection is an approved treatment for newly diagnosed or recurrent high-grade glioma (HGG). The safety and efficacy of this treatment have been established in phase III studies in the United States and Europe.^1,2^ After a multicenter phase I/II study of BCNU wafer implantation,^3^ this treatment was approved in Japan.^4^ BCNU wafers can prolong the survival of initially diagnosed HGG patients.^2,5,6^ These wafers dynamically change various characteristics on magnetic resonance imaging (MRI) between 1 day and 6 months after surgery, including gas promotion in the peri-BCNU wafer area, restricted diffusivity reshaping the silhouette of the wafer surface, contrast enhancement at the rim of the resection cavity, and cerebral edema in the adjacent brain parenchyma.^7–10^ Adverse events with implantation of BCNU wafers were pointed as surgical site infection, cerebrospinal fluid leak, and delayed cerebral edema.^1–6,11^ In several reports, delayed cerebral edema is the most frequent adverse event (22.5–37.5%), and 3–17% of patients with wafer implantation had marked symptoms or required readmission.^2,3,11^ Severe delayed cerebral edema often causes permanent morbidity even with corticosteroid therapy.

We found that some cases of the early experience of BCNU wafer implantation had slight expansion of the high-intensity areas (HIAs) on T_2_-weighted imaging in parenchyma adjacent to the BCNU wafer even in the early postoperative (ep-) MRI (ep-T_2_-HIA expansion), and these cases tended to have delayed cerebral edema. However, association between such ep-T_2_-HIA expansion after BCNU wafer implantation and delayed cerebral edema had not previously been described. We therefore focused on ep-T_2_-HIA expansion and its relation to delayed cerebral edema.

## Materials and Methods

This retrospective study was approved by the ethics committee of our institution (H27–88).

### Study population

This study involved 25 consecutive cases of HGG patients (13 women, 12 men; mean age, 60.4 years; range, 25–79 years) who signed a written informed consent form and underwent removal surgery with BCNU wafer (Gliadel^®^ Wafer, Eisai Co., Tokyo) implantation. The patients were enrolled at our institution between February 2010 and November 2014 (Table 1). In all cases, wafers were placed into the resection cavity after the diagnosis of highly suspected (or undeniable) HGG from intraoperative pathological diagnosis. These 25 patients included 10 patients with initial diagnosis of HGG and 15 patients with recurrent HGG.

For 7 out of 10 initially diagnosed patients, Stupp regimen with temozolomide (TMZ) concomitant with 60 Gy of conventional radiation therapy was used.^12^ For three initially diagnosed patients, only 60 Gy of conventional radiation therapy was used because of several complications including neutropenia and cholecystitis. Seven recurrent patients underwent 5-day maintenance TMZ therapy, one patient who was previously treated only with chemotherapy underwent modified Stupp regimen with TMZ concomitant with proton radiation therapy followed by bevacizumab therapy, and 1 recurrent patient underwent bevacizumab therapy. Other patients were observed without any chemotherapy. The postoperative corticosteroid within 5 postoperative days (PODs) was used for 4 patients and over 6 PODs only for 2 patients. Long-term corticosteroid maintenance and/or bevacizumab therapy were used for 5 patients with delayed cerebral edema. The bevacizumab was used at least 4 weeks after surgery. For 13 patients, neither corticosteroid nor bevacizumab was used for at least 3 postoperative months. In all cases, ep-MRI was performed within 72 h after surgery. Through the evaluation of ep-MRI, patients were divided into two groups: group with ep-T_2_-HIA and without ep-T_2_-HIA. We confirm the incidence of delayed adverse effects in the two groups. Subsequently, the patients were divided into groups with delayed edema and without edema, and various factors were compared between the two groups.

### Image acquisition

In this study, we used two different MR-scanners and protocols for 14 patients treated with an intraoperative MRI (iMRI)-guided surgery and 11 patients treated with a conventional surgery without iMRI. All MRI protocols were implemented by one of the authors (T.M., with 21 years of experience in MRI).

iMRI-guided surgery, preoperative MRI (pre-MRI), and ep-MRI were performed on an iMRI suite (IMRIS VISIUS Surgical Theatre, IMRIS, Winnipeg, Canada) with a modified ceiling-mounted 1.5-T moveable magnet (Espree; Siemens Medical Systems, Erlangen, Germany) with a manufacturer-provided 12-channel head coil for reception and a body coil for transmission. Imaging sequences included three-dimensional (3D) T_1_-weighted magnetization-prepared rapid gradient-echo (MP-RAGE) (section thickness, 1.0 mm; field of view [FOV], 250 mm; echo time [TE], 3.6 ms; repetition time [TR], 1980 ms; inversion time [TI], 1100 ms; flip angle, 15; scan time, 6 min 51 s), 3D T_2_-weighted turbo spin echo (TSE) sequence with variable flip angle echo train (SPACE) (section thickness, 2.0 mm; FOV, 250 mm; TE, 268 ms; TR, 2700 ms; flip angle, 120; scan time, 7 min 34 s), 3D SPACE fluid-attenuated inversion recovery (FLAIR) (section thickness, 2.0 mm; FOV, 250 mm^2^; TE, 282 ms; TR, 4900 ms; TI, 1800 ms; flip angle, 120; scan time, 6 min 34 s), diffusion-weighted imaging (DWI) (section thickness, 5.0 mm; FOV, 230 mm^2^; TE, 94 ms; TR, 5000 ms; b-value, 1000; scan time, 50 s). If a tumor demonstrated contrast enhancement on preoperative images, the 3D T_1_W MP-RAGE sequence was repeated after intravenous administration of gadopentetate dimeglumine (0.1 mmol/kg).

Pre-MRI and ep-MRI for all patients with the conventional surgery without iMRI were performed using a 3.0 T whole-body MR imaging system (Achieva 3.0 T; Philips Healthcare, Best, The Netherlands) with a manufacturer-provided 32-channel head coil for reception and a body coil for transmission. Imaging sequences included 3D T_1_-weighted fast-field echo (T_1_-FFE) sequence (section thickness, 1.0 mm; FOV, 260 mm; TE, 2.3 ms; TR, 6.0 ms; flip angle, 15; scan time, 3 min 58 s), 2D T_2_-weighted TSE sequence (section thickness, 5.0 mm; FOV, 250 mm; TE, 80 ms; TR, 3000 ms; flip angle, 90; scan time, 1 min 47 s), 2D FLAIR sequence (section thickness, 5.0 mm; FOV, 250 mm^2^; TE, 125 ms; TR, 11,000 ms; TI, 2800 ms; flip angle, 90; scan time, 2 min 45 s), DWI sequence (section thickness, 5.0 mm; FOV, 230 mm^2^; TE, 65 ms; TR, 5000 ms; b-value, 1000 s/mm^2^; scan time, 60 s). If the tumor demonstrated contrast enhancement in preoperative studies, the 3D T_1_-FFE sequence was repeated after intravenous administration of gadopentetate dimeglumine (0.1 mmol/kg). Follow-up MRI for all 25 cases were performed using similar sequences on 1.5 T or 3.0 T MRI. The follow-up MRI scans were routinely carried out every 2 months over 6 months and when clinical event occurred.

### Image evaluation and clinical adverse event assessment

Comparing ep-MRI with pre-MRI, early postoperative changes were the gas expansion in a peri-BCNU-wafers area, the ep-T_2_-HIA, and gadolinium (Gd)-enhancement in parenchyma adjacent to BCNU wafers, by evaluating contrast-enhanced T_1_-weighted imaging (T_1_WI), T_2_-weighted imaging (T_2_WI), FLAIR imaging, and DWI. The ep-T_2_-HIA expansion was defined as HIA on T_2_WI of ep-MRI (1) in the parenchyma adjacent to wafers, (2) out of the range of preoperative T_2_-HIA for excluding residual tumor or existing edema, (3) without restricted diffusivity for excluding a postsurgical ischemic lesion, and (4) with the depth of 5 mm or more. For exact detection of ep-T_2_-HIA expansion, iMRI data were also referred to for 14 patients with the iMRI-guided surgery.

To assess whether ep-T_2_-HIA expansion on ep-MRI was associated to various delayed changes, the ratio of delayed cyst enlargement (so-called “bed cyst” formation), delayed cerebral edema, and hydrocephalus were compared between ep-T_2_-HIA expansion group and no expansion group. Adverse effects due to any delayed findings were also evaluated. The delayed cerebral edema was defined as the edema spread to another gyrus beyond the peri-BCNU white matter and the bed cyst was as the major axis of cyst expanded over 20%. The image evaluation was performed independently by two neurosurgeons (E. I. and Y. M., 21 and 11 years of experience in neurosurgery, respectively) with no reference to other clinical data. For assessment of clinical adverse events related to the implantation of BCNU wafers, we used the Common Terminology Criteria for Adverse Events (CTCAE) v.4.0.

To assess factors associated with cerebral edema, the following factors were compared between the delayed and non-delayed edema groups; preoperative data (age, tumor laterality, tumor region, initial or repeated surgery, cyst component, and necrosis), intraoperative data (ventricular opening, extent of removal, and intraoperative pathological diagnosis), ep-MRI data (cavity size, gas in the cavity, DWI HIA around the cavity, enhancement around the cavity, and ep-T_2_-HIA without restricted diffusivity around the cavity), and postoperative pathological data and delayed postoperative data (bed cyst formation, hydrocephalus, adverse effects).

### Data analysis and statistical analysis

All results are presented as absolute number (%) or median (range). Statistical analyses were performed using standard statistical software (SPSS version 21.0 for Mac, SPSS, Chicago, IL, USA). For comparison of early postoperative changes in MRI characteristics Fisher’s exact test was used.

Univariate analysis (Fisher’s exact test) was performed to clarify the relationship between cerebral edema and other individual factors. Factors showing *P* values less than 0.1 by the univariate analysis were included in the multivariate logistic regression analysis. Values of *P* < 0.05 were considered statistically significant.

## Results

### ep-MRI and follow-up MRI characteristics after BCNU wafers implantation

On ep-MRI comparing with pre-MRI, obvious gas was seen in the resection cavity in 23 of 25 BCNU cases (92%). Restricted diffusivity (HIA on DWI) and Gd-enhancement around the cavity were seen in part of the adjacent brain parenchyma in all cases (100%) and 18 cases (72%), respectively. In 9 cases (36%), ep-T_2_-HIA expansion was detected in the parenchyma adjacent to BCNU wafers. In all cases of ep-T_2_-HIA, the FLAIR imaging showed similar high intensity. There was no difference in incidence of the ep-T_2_-HIA expansion in the iMRI-guided surgery group and the conventional surgery group (40% vs. 45%, respectively, *P* = 0.434, Fisher’s direct method).

In the follow-up MRI characteristics, delayed cerebral edema was seen between 4 and 60 days (the peak was between 7 and 101 days) in 12 cases (CTCAE grade 1, n = 7; CTCAE grade 2, n = 2; CTCAE grade 3, n = 3). The bed cyst formation and hydrocephalus were seen in 10 cases (CTCAE grade 1, n = 5; CTCAE grade 2, n = 2; CTCAE grade 3, n = 3), and 3 cases (CTCAE grade 2, n = 1; CTCAE grade 3, n = 2), respectively. Five (50%) of 10 patients with bed cyst, 5 (42%) of 12 patients with delayed cerebral edema, and 4 (57%) of 7 patients both with bed cyst and delayed cerebral edema were symptomatic. As for other adverse effects, mild wound trouble (CTCAE grade 2) was seen only in 2 cases. No patients showed infection or convulsions.

### Factors associated with delayed cerebral edema

**Fig. 1.**
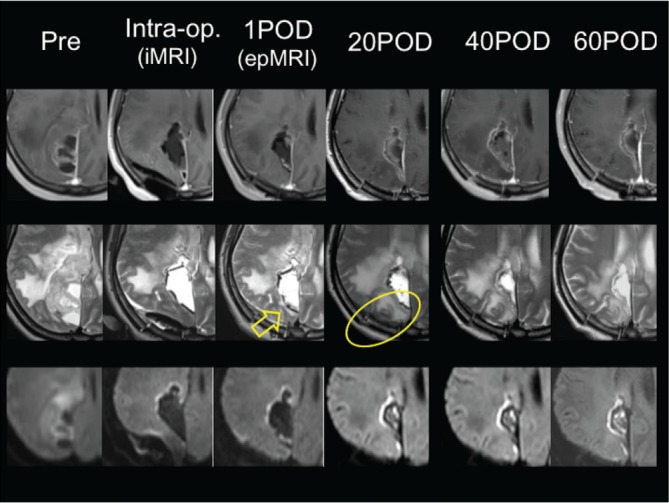
Gadolinium (Gd)-enhanced T_1_-weighted imaging (T_1_WI) (upper images), T_2_WI (center row of images) and diffusion-weighted imaging (DWI) (lower images) of a representative carmustine (BCNU) wafer-implanted case (43 y.o. woman, secondary glioblastoma with an oligodendroglia component) with slight early postoperative high-intensity area on T_2_WI (ep-T_2_-HIA) expansion (a depth of 6.1 mm) in the adjacent parenchymal area without markedly restricted diffusivity (yellow arrow). The T_2_-HIA on early postoperative magnetic resonance imaging (ep-MRI) changes to delayed brain edema 20 days after surgery (yellow circle).

**Fig. 2.**
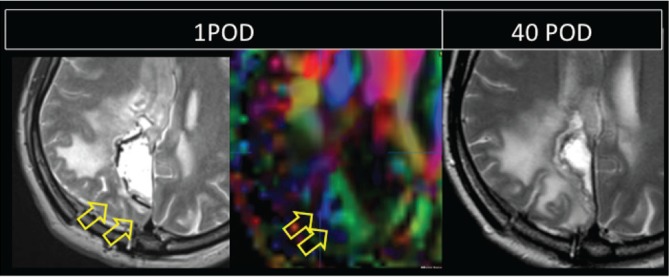
In the same case as Fig. 1, the small high-intensity area on T_2_-weighted imaging (T_2_-HIA) expansion (yellow arrows) on POD 1 (left, T_2_WI) is enlarged on postoperative day (POD) 40 (right) along green fibers (yellow arrows) that indicate anterior-posterior direction in diffusion tensor image color-coded map on POD 1 (middle).

**Fig. 3.**
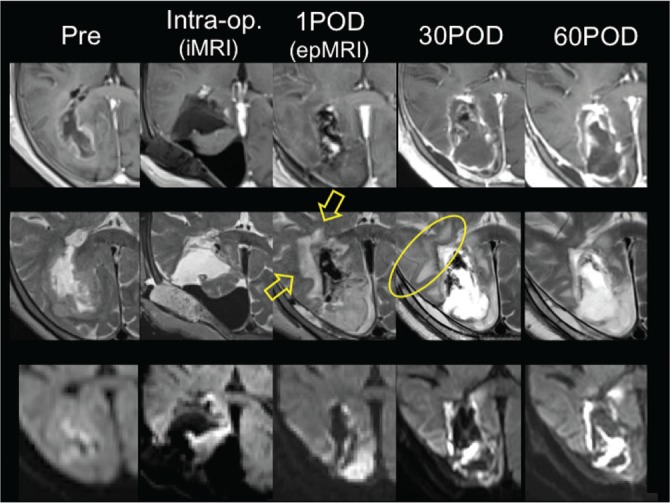
Gadolinium (Gd)-enhanced T_1_-weighted imaging (T_1_WI) (upper), T_2_WI (center) and diffusion-weighted imaging (DWI) (lower) of a representative carmustine (BCNU) wafer-implanted case (76 y.o. woman, initially diagnosed with glioblastoma) with obvious early postoperative high-intensity area on T_2_WI (ep-T_2_-HIA) expansion with a depth of 17.6 mm (yellow arrows) in the adjacent parenchymal area without markedly restricted diffusivity. The ep-T_2_-HIA changes to delayed cerebral edema 30 days after the surgery (yellow circle).

As shown in Table 2, in comparison of 9 cases in the ep-T_2_-HIA group and 16 cases in the non-ep-T_2_-HIA group, delayed cerebral edema was seen in 8 cases (89%) and 4 cases (25%), respectively (*P* = 0.004). The depth of ep-T_2_-HIA expansion from the adjacent resection cavity ranged from 5.8 mm to 17.6 mm with a median length of 7.7 mm. Delayed edema was the only delayed adverse effect associated with ep-T_2_-HIA expansion. Representative MRI in a case with wafer implantation indicating slight ep-T_2_-HIA expansion in the adjacent parenchymal area without markedly restricted diffusivity is shown in Fig. 1. In this case, the T_2_-HIA expansion on POD 1 was enlarged on POD 40 along the white-matter fibers in the diffusion tensor image color-coded map on POD 1 (Fig. 2). A similar case is shown in Fig. 3.

To assess factors associated with cerebral edema, various factors were compared between the delayed and non-delayed edema groups. As shown in Table 3, there was no difference between the two groups in any factor of patient background or intraoperative information. Univariate analysis using various ep-MRI data from the 25 cases showed that the ratio of delayed edema differed significantly between subgroups for removed cavity size (maximal diameter, >40 vs. ≤40 mm; *P* = 0.047) and ep-T_2_-HIA expansion in the adjacent parenchyma with no restricted diffusivity (yes vs. no; *P* = 0.004) (Table 4). We found only tendencies for enhancement around the cavity on ep-MRI (yes vs. no; *P* = 0.073). As shown in Table 5, multivariate analysis using these three factors on ep-MRI showed that the ratio of delayed edema differed significantly between subgroups only for ep-T_2_-HIA expansion (*P* = 0.0208, odds ratio = 28.7).

In other various factors except ep-MRI and adverse effects, we found only tendencies for negative staining for isocitrate dehydrogenase 1 (*IDH-1*) mutation (*P* = 0.074), and delayed hydrocephalus (*P* = 0.096). Univariate analysis showed that the ep-T_2_-HIA expansion was not associated with the *IDH-1* status or delayed hydrocephalus. There was no relation between the delayed edema and other various pathological data including existence of oligo component (yes vs. no, *P* = 0.999), MIB-1 index (≥25 vs. <25, *P* = 0.238), mitosis (≥5/10 high-power-field (HPF) vs. <5/10 HPF, *P* = 0.999), O^6^-methylguanine-DNA methyltransferase promotor by methylation-specific polymerase chain reaction (methylated vs. unmethylated, *P* = 0.691), p53 stain by immunohistochemistry (<10% vs. ≥10%, *P* = 0.697), pathological diagnosis using permanent section (GBM vs. others, *P* = 0.111), and final clinical diagnosis (GBM vs. others, *P* = 0.999). There was no difference in incidence of the delayed edema between the iMRI-guided surgery group (50%) and the conventional surgery group (43% vs. 55%, *P* = 0.695, Fisher’s direct method).

## Discussion

In our study, gas, ep-T_2_-HIA expansion and contrast enhancement on T_1_WI at the rim of the resection cavity were frequently observed after BCNU implantation. Previous studies of MRI characteristics after implantation of BCNU wafers have indicated dynamic changes near the wafers on T_1_WI and T_2_WI from POD 1 to 6 months postoperatively, including gas expansion in the peri-BCNU-wafer area, restricted diffusivity reshaping the silhouettes of wafer surfaces, contrast enhancement at the rim of the resection cavity, and cerebral edema in the adjacent brain parenchyma.^7–10^ Restricted diffusivity, contrast enhancement, and T_2_-HIA expansion at the rim of the resection cavity can already be found on POD 1.^10^ Linear enhancement on ep-MRI has generally been considered potentially attributable to contrast “leakage” at the resection margins or to small amounts of residual glial tumor.^13^ To evaluate the surgical outcomes and response to subsequent chemoradiotherapy, the RANO working group recommended that postsurgical MRI should be completed within 72 h.^14,15^ However, the influence of BCNU wafers was already apparent on ep-MRI, as shown in the present experiments. When assessing the extent of resection or response to subsequent chemoradiotherapy, we have to keep in mind that ep-MRI may already be affected by the BCNU wafers. In particular, hyperintense changes on T_2_WI (ep-T_2_-HIA expansion) and contrast enhancement at the rim of the resection cavity on Gd-enhanced T_1_WI were difficult to distinguish from the residual tumor. iMRI might be recommended to estimate the extent of resection, because implanted BCNU wafers affected iMRI data less than ep-MRI images in our cases (detailed data not shown).

We observed ep-T_2_-HIA expansion in 36% of BCNU implantation cases and delayed cerebral edema in 12 cases (48%). The high incidence of edema is unsurprising, because various studies of BCNU wafer implantation have shown a high frequency of edema (22.5–37.5%).^2,3,11^ On the other hand, marked edema (CTCAE grade 3 or more) was seen in only 12% of cases, comparable to previous studies (3–17%).^3,6,11^ Factors associated with delayed cerebral edema remain unclear. In the present study, delayed edema was associated with maximal diameter of the resection cavity (≤40 mm) and ep-T_2_-HIA expansion. A previous study showed that patients who developed cerebral edema were frequently affected by delayed bed cyst enlargement (so-called “bed cyst”),^7^ although only a tendency toward associations with bed cysts was seen in our study.

Median depth for ep-T_2_-HIA expansion from the adjacent resection cavity was 7.7 mm, and ranged from 5.8 to 17.6 mm. In a study of pharmacokinetics with interstitial delivery of BCNU using cynomolgus monkeys, high drug concentrations were seen at 6.1 ± 2.9 mm on POD 1 and significant levels of the drug were also present up to ∼5 cm from the polymer implant.^16^ In the resection cavity wall, which consists of traumatized brain parenchyma, “bulk flow” is thought to be the predominant mechanism of BCNU distribution, rather than diffusion, leading to high penetration distances for BCNU.^17,18^ We speculate that T_2_-HIA expansion on ep-MRI indicates that BCNU flows via “bulk flow” through the exposed face of the white matter tracts (as shown in Fig. 2), resulting in delayed cerebral edema in a large parenchymal area, and the area of markedly restricted diffusivity indicates infarction due to high drug concentrations via diffusion.

In our study, small resection cavity was also a predictive factor of delayed edema in univariate analysis, although multivariate analysis did not show statistical relationship between them. Since number of the implanted wafers was 6 to 8 regardless of the cavity size, reaction of parenchymal lesion by wafers in small cavity cases may be stronger than in large cavity cases. We believe that the delayed edema is a result of antitumor and/or inflammatory reaction, resulting in a favorable outcome for the patient, although significant difference was not observed in overall survival between edema and non-edema groups (*P* = 0.384).

There were some limitations to our study. The small number of cases may have some bearing on the results. We evaluated pre-MRI and ep-MRI by different equipment and protocols in the iMRI-guided surgery group and conventional surgery group, which also might affect the results, although there was no difference in incidence of ep-T_2_-HIA expansion or delayed edema between the two groups. Moreover, heterogeneous population of HGG might make it difficult to evaluate with precision whether or not the delayed edema affects the outcome in such patients, and further analysis using larger number of cases is necessary. The ep-T_2_-HIA expansion with no restricted diffusivity can be caused by some factors including cerebral contusion and venous edema, independently of wafer implantation. In our preliminary study, only minimal ep-T_2_-HIA expansion with no restricted diffusivity was observed in all post- removal cases without wafer implantation, and the depth (median, 0 mm; range, 0–3.4; n = 9) was significantly lower than that (median, 6.5 mm; range, 0–11.6; n = 9) in wafer implanted cases (*P* = 0.0200, Mann–Whitney U test, detailed data not shown). Nevertheless, we should keep in mind that the ep-T_2_-HIA expansion is not a specific finding after wafer implantation.

## Conclusion

Delayed edema was detected in 48% of cases after BCNU wafer implantation and in 42% of the cases the patients had corresponding symptoms. Delayed edema was associated with the expansion of ep-T_2_-HIAs, as well as the maximal diameter of the resection cavity (≤40 mm) in univariate analysis. Multivariate analysis showed that ep-T_2_-HIA expansion was the only independent factor among ep-MRI data associated with delayed edema.

## Figures and Tables

**Table 1.** High-grade glioma (HGG) patients who underwent carmustine (BCNU) wafers implantation

			BCNU cases
Number			25
Mean age			60.4
Sex (M/F)			12/13
Initial or repeated surgery (initial/repeated)			10/15
Extent of removal, median (range)			99% (50–100)
Number of BCNU wafers implanted, median (range)			8 (6–8)
Preoperative clinical diagnosis (HGG)			25

Postoperative clinical diagnosis		AA	3
		AO/AOA	4
		GBM	15
		GBMO	3

Postoperative treatments	Initial surgery cases	CRT + TMZ	7
		CRT	3

	Repeated surgery cases	Maintenance TMZ	7
		PT + TMZ + BEV	1
		BEV	1
		Observation	6

AA, anaplastic astrocytoma; AO, anaplastic oligodendroglioma; AOA, anaplastic oligoastrocytoma; BEV, bevacizumab; CRT, conventional radiation therapy; F, female; GBM, glioblastoma; GBMO, glioblastoma with oligodendroglial component; M, male; PT, proton therapy; TMZ, temozolomide

**Table 2.** Statistical analysis comparing follow-up magnetic resonance imaging (MRI) to early postoperative MRI (ep-MRI) in 25 carmustine (BCNU) wafer-implanted patients with or without early postoperative high-intensity area on T_2_-weighted imaging (ep-T_2_-HIA) expansion

	With ep-T_2_-HIA n = 9	Without ep-T_2_-HIA n = 16	*P* value*
Any delayed findings on follow-up MRI, n (%)	9 (100)	6 (38)	0.003
Bed cyst formation on follow-up MRI, n (%)	5 (56)	5 (31)	0.397
Cerebral edema on follow-up MRI, n (%)	8 (89)	4 (25)	0.004
Hydrocephalus on follow-up MRI, n (%)	2 (22)	1 (6)	0.530
CTCAE grade 2 or more adverse effects due to any delayed findings (yes), n (%)	3 (33)	4 (25)	0.673

* Fisher’s direct method; CTCAE, Common Terminology Criteria for Adverse Event v.4.0

**Table 3.** Univariate analysis to assess preoperative and intraoperative factors associated with cerebral edema in 25 carmustine (BCNU) wafer-implanted patients

Factors	Edema		Univariate (Fisher’s direct method)
	
	Delayed edema	Non-delayed edema	*P* value
	
Preoperative data	n = 12	n = 13	
Age ≥60 years, n (%)	10 (83%)	7 (54%)	0.202
Laterality (right), n (%)	7 (58%)	8 (62%)	0.999
Region of tumor (frontal), n (%)	5 (42%)	9 (69%)	0.238
Initial surgery (yes), n (%)	7 (58%)	3 (23%)	0.111
Cyst component on preop. MRI (yes), n (%)	4 (33%)	3 (23%)	0.673
Necrosis on preop. MRI (yes), n (%)	9 (75%)	11 (86%)	0.645
Intraoperative data			
Ventricular opening (no), n (%)	9 (75%)	6 (46%)	0.226
Extent of removal (partial), n (%)	4 (33%)	1 (8%)	0.160
HGG by intraoperative pathological diagnosis, n (%)	6 (50%)	8 (62%)	0.680

HGG, high-grade glioma; MRI, magnetic resonance imaging; preop., preoperative

**Table 4.** Univariate analysis to assess postoperative factors associated with delayed cerebral edema

Factors	Edema		Univariate (Fisher’s direct method)
	
	Delayed edema	Non-delayed edema	*P* value
		
Early postoperative MRI data	n = 12	n = 13	
Cavity size ≤40 mm, n (%)	9 (75%)	4 (31%)	0.047
Gas in the cavity (yes), n (%)	11 (92%)	12 (92%)	0.999
DWI HIA around the cavity (yes), n (%)	12 (100%)	13 (100%)	0.999
Enhancement around the cavity (yes), n (%)	11 (92%)	7 (54%)	0.073
ep-T_2_-HIA without restricted diffusivity around the cavity (yes), n (%)	8 (67%)	1 (8%)	0.004
Postoperative pathological data (please see the text about other pathological data)			
MGMT promoter by MSPCR (methylated), n (%) <n = 22>	4 (40%)	6 (50%)	0.691
IHC staining for *IDH-1*-R132H mutations (negative), n (%) <n = 22>	9 (90%)	6 (50%)	0.074
Delayed postoperative MRI data			
Bed cyst formation (yes), n (%)	7 (58%)	3 (23%)	0.111
Hydrocephalus (yes), n (%)	3 (25%)	0 (0%)	0.096
CTCAE due to any delayed change (≥grade 2), n (%)	6 (50%)	1 (8%)	0.030
Peak of delayed edema from surgery, median days count (range)	28 (7–101)	–	–
Outcome			
Alive after wafer implantation (alive case%, mOS [mos])	83%, n.r.	54%, 23 mos	0.384**

CTCAE, Common Terminology Criteria for Adverse Event v.4.0; DWI, diffusion-weighted imaging; ep-T_2_-HIA, early postoperative high-intensity area on T_2_-weighted imaging; HIA, high-intensity area; *IDH*-1, isocitrate dehydrogenase 1; IHC, immunohistochemical; mOS, median overall survival from wafers implantation; n.r., not reached; MGMT, O^6^-methylguanine-DNA methyltransferase; MSPCR, methylation- specific polymerase chain reaction; MRI, magnetic resonance imaging

** Logrank test

**Table 5.** Logistic regression analysis to assess early postoperative magnetic resonance imaging (MRI) data associated with delayed cerebral edema

Factors	Multivariate logistic regression analysis		
	*P* value	Odds ratio	Range
	
Cavity size ≤40 mm	0.0712	11.9	(0.807–167)
Enhancement around the cavity	0.0909	18.2	(0.629–528)
ep-T_2_-HIA without restricted diffusivity around the cavity	0.0208	28.7	(1.67–496)

ep-T_2_-HIA, early postoperative high-intensity area on T_2_-weighted imaging
